# Supplementary material for: Evidence supported by Mendelian randomization: impact on inflammatory factors in knee osteoarthritis
Source: Front Med (Lausanne). 2024 May 28;11:1382836. doi: 10.3389/fmed.2024.1382836 (PMC11165061; doi:10.3389/fmed.2024.1382836)
Supplement: Supplementary file 1 [file Data_Sheet_1.ZIP › data/GCST90274806_5e6/806.leaveoneout.pdf]

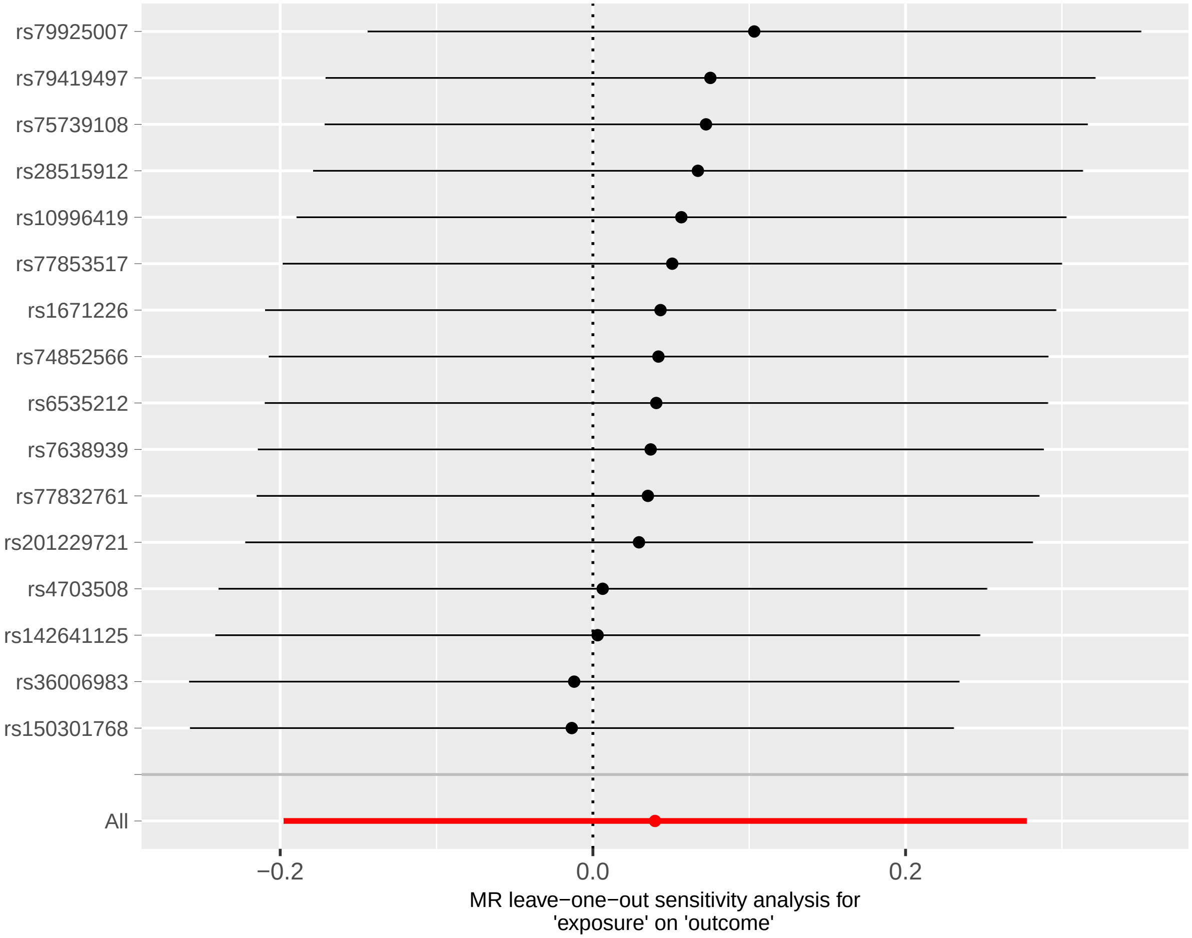
rs79925007
rs79419497
rs75739108
rs28515912
rs10996419
rs77853517
rs1671226
rs74852566
rs6535212
rs7638939
rs77832761
rs201229721
rs4703508
rs142641125
rs36006983
rs150301768
All
−0.2
0.0
0.2
MR leave−one−out sensitivity analysis for
'exposure' on 'outcome'
